# Temporal trends in the prevalence of metabolic syndrome among middle-aged and elderly adults from 2011 to 2015 in China: the China health and retirement longitudinal study (CHARLS)

**DOI:** 10.1186/s12889-021-11042-x

**Published:** 2021-06-02

**Authors:** Bo Liu, Guanqun Chen, Ruijie Zhao, Dan Huang, Lixin Tao

**Affiliations:** 1 Department of Pharmacy, Beijing Xiaotangshan Hospital, Beijing, 102211 China; 2 Department of Neurology, Xuanwu Hospital of Capital Medical University, Beijing, 100053 China; 3 Department of Radiology, Peking Union Medical College Hospital, Chinese Academy of Medical Sciences, Beijing, 100730 China; 4 Department of Development coordination office, Beijing Xiaotangshan Hospital, Beijing, 102211 China; 5 Department of Epidemiology and Health Statistics, School of Public Health, Capital Medical University, Beijing, 100069 China; 6 Beijing Municipal Key Laboratory of Clinical Epidemiology, Beijing, 100069 China

**Keywords:** Metabolic syndrome, Prevalence, Trends, Epidemiology, China

## Abstract

**Background:**

Metabolic syndrome (MetS) is a major risk factor for cardiovascular diseases. The objective of the study was to evaluate the updated prevalence of MetS and provide a comprehensive illustration of the possible temporal changes in MetS prevalence in China from 2011 to 2015.

**Methods:**

The data for this study are from the 2011 and 2015 waves of the China Health and Retirement Longitudinal Study (CHARLS). CHARLS is a nationally representative survey targeting populations aged 45 and above from 28 provinces in mainland China. A total of 11,847 and 13,013 participants were eligible for data analysis at the two time points.

**Results:**

The estimated prevalence of MetS in 2015 was 20.41% (95% CI: 19.02–21.8%) by the National Cholesterol Education Program (NCEP) Expert Panel on Detection, Evaluation, and Treatment of High Blood Cholesterol in Adults (ATP III) criteria, 34.77% (95% CI: 33.12–36.42%) by the International Diabetes Federation (IDF) criteria, 39.68% (95% CI: 37.88–41.47%) by the revised ATP III criteria, and 25.55% (95% CI: 24.19–26.91%) by the Chinese Diabetes Society (CDS) criteria. The prevalence was higher among women and elderly adults and in urban and northern populations. Furthermore, the trends in the prevalence decreased significantly between 2011 and 2015 by the ATP III, revised ATP III and CDS criteria. However, trends increased significantly from 2011 to 2015 by the IDF criteria.

**Conclusions:**

A higher prevalence of MetS is found in those who reported being middle aged and elderly, women, residing in northern China or living in urban areas. Additionally, temporal changes in the prevalence of MetS varied according to different criteria. Increased attention to the causes associated with populations who have higher levels of MetS is warranted.

**Supplementary Information:**

The online version contains supplementary material available at 10.1186/s12889-021-11042-x.

## Background

Metabolic syndrome (MetS) is a constellation of metabolic disturbances, including abdominal obesity, hypertension, dyslipidemia, and elevated plasma glucose [1, 2]. Studies have confirmed that the syndrome is associated with an increased risk of cardiovascular disease and other diseases [3–6]. In light of the substantial burden of cardiovascular disease in China [7], changes in the prevalence of MetS over time play an important reference and guidance role in decisions and implementation of public health measures regarding the control of cardiovascular disease.

Several nationally representative studies have provided evidence for the prevalence of MetS in the Chinese population. According to data from the International Collaborative Study of Cardiovascular Disease in ASIA (InterASIA), a cross-sectional study enrolled 15,540 Chinese adults aged 35–74 years in 2000, and the prevalence of MetS was 13.7% based on definition of the revised ATP III criteria [8]. The China Health and Nutrition Survey (CHNS) conducted in 2009, which is a longitudinal cohort study started in 1989 including a total of 7488 Chinese adults (age ≥ 18 years), declared that the overall age-standardized prevalence of MetS was 21.3% according to the revised ATP III criteria [9]. The 2010 China Noncommunicable Disease Surveillance (CNCDS) included 98,658 Chinese adults (aged ≥18 years) in 2010 and reported that the prevalence of MetS was 33.9% by the revised ATP III criteria [10]. Moreover, the China National Stroke Prevention Project (CSPP) survey in 2014–2015 estimated that the prevalence of MetS was 18.4% based on data from 109,551 participants by the ATP III criteria [11]. The results of these national studies showed a high prevalence of MetS, which has become an important public health problem in China. However, it may be inappropriate to directly compare the results of these studies to reflect the possible temporal changes in MetS prevalence due to cross-sectional design, different sampling methods, inconsistent measurement of indicators, age range of participants and the definitions of MetS adopted.

In this study, we used data from the China Health and Retirement Longitudinal Study (CHARLS) survey to describe the latest prevalence of MetS based on four different diagnostic criteria and then provide a comprehensive illustration of the possible temporal changes in MetS prevalence in China from 2011 to 2015.

## Methods

### Study design and population

Data for this study were obtained from the two waves of the CHARLS survey conducted by the National School of Development at Peking University in 2011 and 2015. CHARLS is a nationally representative survey targeting populations aged 45 and above from 450 villages or communities in 150 counties or districts in 28 provinces of mainland China that provides a wide range of demographic, socioeconomic status and health condition variables. CHARLS participants were followed every 2 years using a face-to-face computer-assisted personal interview (CAPI). The baseline wave for the study was conducted between June 2011 and March 2012 and involved 17,708 respondents with a response rate of 80.5%. The research team collected blood samples from 11,847 individuals. The third wave of the study was conducted in 2015 and involved 20,284 respondents with a response rate of 87%. A total of 13,013 respondents provided venous blood. The details of the sampling design of this survey were previously described in an earlier publication [12].

The medical ethics committee approved the CHARLS study, and all interviewees were required to sign informed consent. Ethics approval for the data collection in CHARLS was obtained from the Biomedical Ethics Review Committee of Peking University (IRB00001052–11015). Ethics approval for the use of CHARLS data was obtained from the University of Newcastle Human Research Ethics Committee (H-2015-0290).

### Data collection

At each county or district unit, trained staff collected data according to a standard protocol in respondents’ homes and local Community or township Healthcare Center and the County Center for Disease Prevention and Control (CDC). A questionnaire based on a face-to-face CAPI was used to comprehensively record social, economic, and health circumstances of participants, such as measures of health, socio-economic factors and health related behaviors and health outcomes, childhood circumstances and community environment, cognitive and physical health, current economic positions, social and family support, health insurance, and health-care utilization. Anthropometric measurements were performed by the interviewers who carried equipment into respondents’ households. Systolic and diastolic blood pressure was measured three times in the left arm with an automated electronic device (OMRON Model HEM-7112, Omron Company), with a 45 s interval between each measurement. The mean of the three measurements was used for analysis. Waist circumference (WC) was measured in a standing position at the level of the umbilicus using a soft tape measure. The participants were instructed to not smoke, eat, drink, chew gum or brush their teeth when measuring.

After completing the questionnaires and measurements, 8 ml fasting blood specimens were collected by trained nurses after an overnight fasting of at least 8 h at township hospitals or the local office of the CDC. The blood sample was separated into plasma and buffy coat and then was immediately shipped back to Beijing at − 20 °C and was stored at the Chinese CDC at − 70 °C until assay at the laboratory of Capital Medical University. Triglyceride (TG) and high-density lipoprotein cholesterol (HDL-C) levels were examined using an enzymatic colorimetric test. Blood glucose levels were assessed using the glucose oxidase method.

### Quality control

Quality control measures were implemented during the fieldwork based on CAPI. Data verification was carried out by CHARLS headquarters. In addition, the first two visits of all interviewers were recorded to verify that the procedures executed were correct. If the recording could not be carried out due to technical problems or other reasons, staff from CHARLS headquarters would call these households for a phone check.

### Definition and diagnostic criteria

MetS was defined according to four different definitions, including National Cholesterol Education Program (NCEP) Expert Panel on Detection, Evaluation, and Treatment of High Blood Cholesterol in Adults (ATP III) criteria [13], criteria from the International Diabetes Federation (IDF) criteria [14], revised ATP III criteria for Asian populations [15], and Chinese Diabetes Society (CDS) criteria [16]. The details are shown in Table 1.

**Table 1:** Diagnosis criteria of metabolic syndrome used in the current study

	ATP III criteria	IDF criteria	Revised ATP III criteria	CDS criteria
To be identified as MetS	Any three or more of the following five components	Central obesity plus any two other factors	Any three or more of the following five components	Any three or more of the following five components
Central obesity (waist circumference)				
Men	>102 cm	≧90 cm for Chinese men	≧90 cm for Asian men	≧90 cm
Women	>88 cm	≧80 cm for Chinese women	≧80 cm for Asian women	≧85 cm
Elevated TG	≧1.70 mmol/L (150 mg/dL)	≧1.70 mmol/L (150 mg/dL) mg/dL or specific treatment for this lipid abnormality	≧1.70 mmol/L (150 mg/dL) or drug treatment for elevated TG	≧1.70 mmol/L(150 mg/dL)
Low HDL-C				
Men	<1.04 mmol/L (40 mg/dL)	<1.04 mmol/L (40 mg/dL) in males or specific treatment for this lipid abnormality	<1.04 mmol/L (40 mg/dL) in men or drug treatment for reduced HDL-C	<1.04 mmol/L(40 mg/dL)
Women	<1.29 mmol/L (50 mg/dL)	<1.29 mmol/L (50 mg/dL) in females or specific treatment for this lipid abnormality	<1.29 mmol/L (50 mg/dL) in women or drug treatment for reduced HDL-C	<1.04 mmol/L(40 mg/dL)
Elevated BP	≧130/85 mmHg	SBP ≧130 or DBP ≧85 mmHg, or treatment of previously diagnosed hypertension	≧130 mmHg SBP or ≧85 mmHg DBP or on antihypertensive drug treatment in a patient with a history of hypertension	SBP ≧130 or DBP ≧85 mmHg, previously diagnosed hypertension
Elevated FPG	≧6.1 mmol/L (110 mg/dL)	≧5.6 mmol/L (100 mg/dL), or previously diagnosed type 2 diabetes	≧5.6 mmol/L (100 mg/dL) or drug treatment for elevated glucose	≧6.1 mmol/L, 2 h plasma glucose ≧7.8 mmol/L, or previously diagnosed diabetes

*Abbreviations*: *MetS* metabolic syndrome, *TG* triglycerides, *HDL-C* high-density lipid cholesterol, *BP* blood pressure, *SBP* systolic blood pressure, *DBP* diastolic blood pressure, *FPG* fasting plasma glucose

### Statistical analysis

Initially, we calculated the prevalence of MetS for the overall population and different subgroups by diagnostic criteria, sex, residence (rural or urban areas), marital status, six age groups (45–50, 51–55, 56–60, 61–65, 66–70, 71 years and older), six regions (Southwest, South-Central, East, Northwest, North, and Northeast China), and components (central obesity, elevated TG, reduced HDL-C, elevated fasting plasma glucose and high blood pressure) in 2011 and 2015, respectively. To adjust for the non-response rate, sample weight for the blood data was used, calculated from a logistic regression in these surveys. Considering different demographic factors of ages and genders in these two surveys, we used the Logistic regression model to calibrate the prevalence. The model expression is

$$\ln \left(\frac{P}{1-P}\right)=\alpha +{\beta}_1{x}_1+{\beta}_2{x}_2+\dots +{\beta}_n{x}_n$$

$$P=\frac{e^{\alpha +{\beta}_1{x}_1+{\beta}_2{x}_2+\dots +{\beta}_n{x}_n}}{1+{e}^{\alpha +{\beta}_1{x}_1+{\beta}_2{x}_2+\dots +{\beta}_n{x}_n}}$$

Further, the prevalence was compared between 2011 and 2015 to reflect temporal trends. The *Chi-square* test was used for statistically testing categorical variables such as sex, residence and marital status. The *P* trend was displayed in the variables of age group and region. Logistic regression was performed to compare the prevalence of MetS in 2011 and 2015, adjusting for demographic factors like age level, sex, marital status, residence and region.

All analyses were completed in SAS statistical software Version 9.4 (SAS Institute Inc., Cary, North Carolina, USA), where all statistical significance was recorded at the 0.05 level and *P* values were two-sided.

## Results

### Characteristics of the study participants in 2011 and 2015

A total of 11,847 and 13,013 subjects were ultimately included in 2011 and 2015, respectively. There was no significant difference in the distribution of sex and marital status. However, significant differences were foundinr the distribution of age group, residence and region. The frequency distributions of elderly subjects in urban areas and in the east in 2015 were significantly higher than those in 2011. Table 2 shows the details of the sample demographics.

**Table 2:** Demographic characteristics between two waves in 2011 and 2015

	Baseline wave in 2011 (11847)	Third wave in 2015 (13013)	*P* value
Sex, n (%)			0.4989
Female	6336 (53.48%)	7019 (53.94%)	
Male	5511 (46.52%)	5994 (46.06%)	
Age group, n (%)			< 0.0001
45–50	2472 (20.87%)	2143 (16.47%)	
51–55	1914 (16.16%)	2092 (16.08%)	
56–60	2418 (20.41%)	2071 (15.91%)	
61–65	1886 (15.92%)	2357 (18.11%)	
66–70	1288 (10.87%)	1741 (13.38%)	
≥ 71	1868 (15.77%)	2609 (20.05%)	
Marital Status, n (%)			0.909
Married^a^	9787 (82.62%)	10,744 (82.56%)	
Abnormal Status^b^	2059 (17.38%)	2269 (17.44%)	
Residence, n (%)			0.0128
Rural	7529 (63.56%)	8007 (62.03%)	
Urban	4317 (36.44%)	4902 (37.97%)	
Region, n (%)			< 0.0001
North-east	847 (7.15%)	938 (7.27%)	
North	1675 (14.14%)	1676 (12.98%)	
North west	955 (8.06%)	1022 (7.92%)	
East	3634 (30.68%)	4359 (33.76%)	
South-central	2661 (22.46%)	2840 (21.99%)	
South-west	2074 (17.51%)	2077 (16.08%)	

^a^married with spouse present; ^b^married but not living with spouse temporarily for reasons such as work, separated, divorced, widowed and never married

### Prevalence of metabolic syndrome in 2011 and 2015

**Fig. 1:**
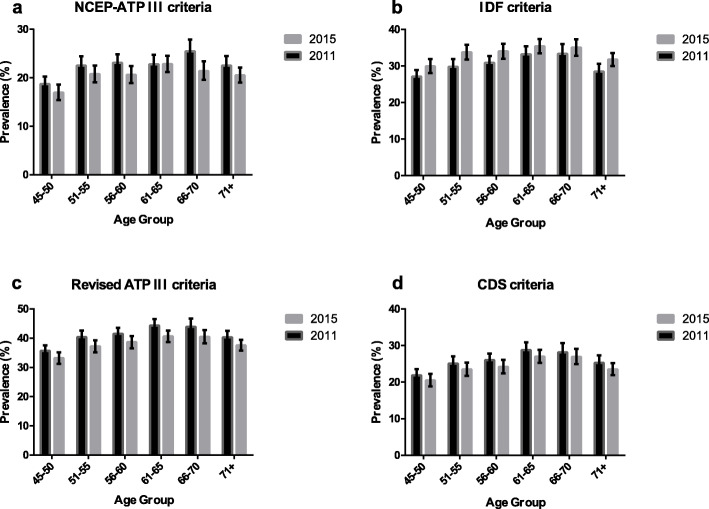
Age-specific prevalence of metabolic syndrome based on different definitions in 2011 and 2015. Figure a, b, c, and d shows the prevalence of metabolic syndrome between different age groups based on ATP III, IDF, revised ATP III and CDS criteria, respectively. Dotted line represents the baseline wave and a solid line represents the third wave

**Fig. 2:**
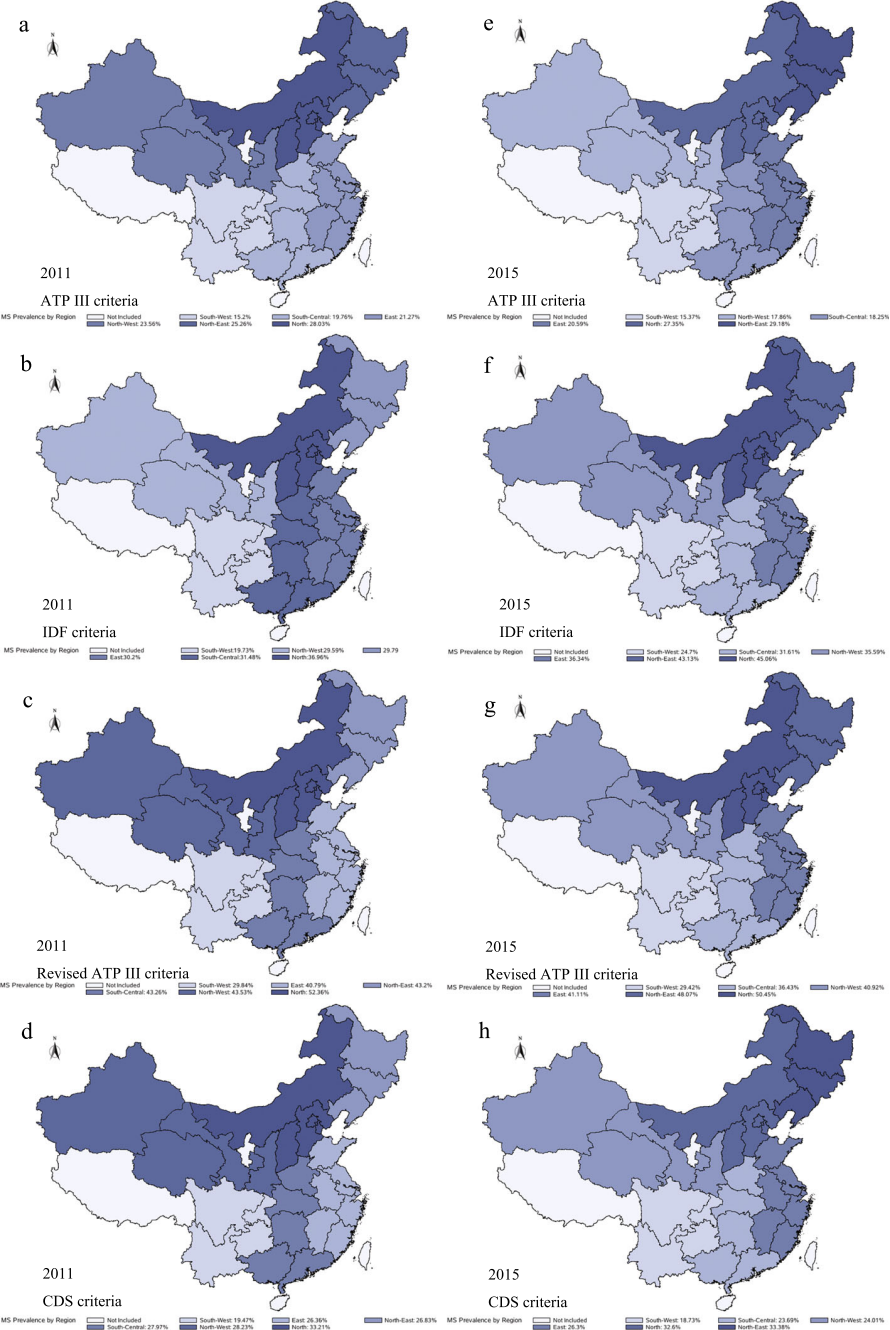
Region-specific prevalence of metabolic syndrome based on different definitions in 2011 and 2015. Figure a, b, c, and d indicate the prevalence of metabolic syndrome in different regions in 2011 based on ATP III, IDF, revised ATP III and CDS criteria, respectively. Figure e, f, g, and h indicates the prevalence of metabolic syndrome in different regions in 2015 based on ATP III, IDF, revised ATP III and CDS criteria, respectively. Different colors represent different regions of China. Individuals living in Hainan, Ningxia, Taiwan, and Tibet were not included in the surveys

The top half of Table 3 displays the total-, sex-, marital status- and residence-specific prevalence of MetS based on different definitions among Chinese middle-aged and elderly adults in 2011. Overall, the prevalence of MetS was 21.18% (95% CI: 19.48–22.88%), 29.57% (95% CI: 27.52–31.61%), 41.44% (95% CI: 39.24–43.65%) and 26.66% (95% CI: 25.09–28.22%) based on definitions of ATP III, IDF, revised ATP III and CDS criteria, respectively. The prevalence in women was significantly higher than that in men, and the prevalence in the urban population was significantly higher than that in the rural population regardless of the definitions used. There was no difference in prevalence between marital status except that the prevalence in married individuals was significantly higher than that in abnormal status using CDS criteria. Notably, the prevalence increased with age (Fig. 1). The top half of Additional file 1: Table S1 provides specific data. In addition, the prevalence was remarkably higher in the north population than in the south population regardless of the definitions used (Fig. 2, Panels a, b, c, and d). For exact data, see the top half of Additional file 1: Table S2.

**Table 3:** Total, sex-, marital status- and residence-specific prevalence of metabolic syndrome based on different definitions in 2011 and 2015

	n	ATP III criteria %(95% CI)	IDF criteria %(95% CI)	Revised ATP III criteria %(95% CI)	CDS criteria %(95% CI)
2011					
Total	11,846	21.18 (19.48–22.88)	29.57 (27.52–31.61)	41.44 (39.24–43.65)	26.66 (25.09–28.22)
Sex					
Male	5502	14.12 (12.55–15.7)	18.4 (16.59–20.22)	32.54 (30.4–34.68)	24.62 (22.68–26.57)
Female	6336	27.72 (25.45–29.98)	39.9 (37.11–42.68)	49.69 (46.24–53.14)	28.56 (26.65–30.48)
*P* value		< 0.0001	< 0.0001	< 0.0001	0.0007
Marital Status					
Married	9787	20.89 (19.54–22.24)	30.04 (28.16–31.92)	42.06 (39.76–44.36)	27.45 (25.79–29.1)
Abnormal status	2059	22.52 (19.51–25.53)	27.61 (24.37–30.85)	38.87 (34.87–42.86)	23.32 (20.29–26.35)
*P* value		0.3197	0.2049	0.1684	0.0203
Residence					
Rural	7529	19.26 (17.95–20.57)	26.14 (24.45–27.83)	35.64 (33.8–37.48)	22.06 (20.62–23.5)
Urban	4317	23.22 (20.47–25.97)	33.2 (30.16–36.24)	47.61 (44.38–50.84)	31.54 (28.56–34.52)
*P* value		0.0099	< 0.0001	< 0.0001	< 0.0001
2015					
Total	11,013	20.41 (19.02–21.8)	34.77 (33.12–36.42)	39.68 (37.88–41.47)	25.55 (24.19–26.91)
Sex					
Male	5991	12.12 (10.6–13.64)	25.57 (23.83–27.32)	31.96 (29.91–34.01)	25.94 (23.95–27.93)
Female	7019	28.01 (26.46–29.56)	43.2 (41.14–45.25)	46.74 (44.72–48.76)	25.2 (23.78–26.62)
*P* value		< 0.0001	< 0.0001	< 0.0001	0.5052
Marital Status					
Married^a^	10,744	20.1 (18.76–21.44)	34.6 (33.02–36.18)	39.42 (37.73–41.11)	25.81 (24.44–27.18)
Abnormal status^b^	2269	21.97 (19.79–24.15)	35.63 (33.19–38.07)	40.96 (38.41–43.51)	24.3 (22.09–26.52)
*P* value		0.1407	0.4807	0.3175	0.2583
Residence					
Rural	8008	17.3 (16.15–18.44)	28.39 (26.98–29.81)	32.84 (31.39–34.29)	20.42 (19.17–21.68)
Urban	4904	23.59 (21.23–25.96)	41.27 (38.6–43.94)	46.65 (43.59–49.7)	30.78 (28.55–33.01)
*P* value		< 0.0001	< 0.0001	< 0.0001	< 0.0001

^a^married with spouse present; ^b^married but not living with spouse temporarily for reasons such as work, separated, divorced, widowed and never married

The total-, sex-, marital status- and residence-specific prevalence of MetS based on different definitions among Chinese middle-aged and elderly adults in 2015 were summarized in the bottom half of Table 3. Overall, the prevalence of MetS was 20.41% (95% CI: 19.02–21.8%), 34.77% (95% CI: 33.12–36.42%), 39.68% (95% CI: 37.88–41.47%) and 25.55% (95% CI: 24.19–26.91%) based on definitions of ATP III, IDF, revised ATP III and CDS criteria, respectively. The prevalence in women was significantly higher than that in men, except the prevalence was similar using CDS criteria. In addition, the prevalence in urban areas was significantly higher than that in rural areas regardless of the criteria applied. Interestingly, there was no difference in prevalence between marital status. Similar to the description in 2011, the prevalence increased with age except for the prevalence based on IDF criteria (Fig. 1, the numeric data in the bottom half of Additional file 1: Table S1). The prevalence was higher in the northern population than in the southern population regardless of the definitions used (Fig. 2, Panels e, f, g, and h). Data are shown in the bottom half of Additional file 1: Table S2.

### Prevalence of MetS components in 2011 and 2015

**Fig. 3:**
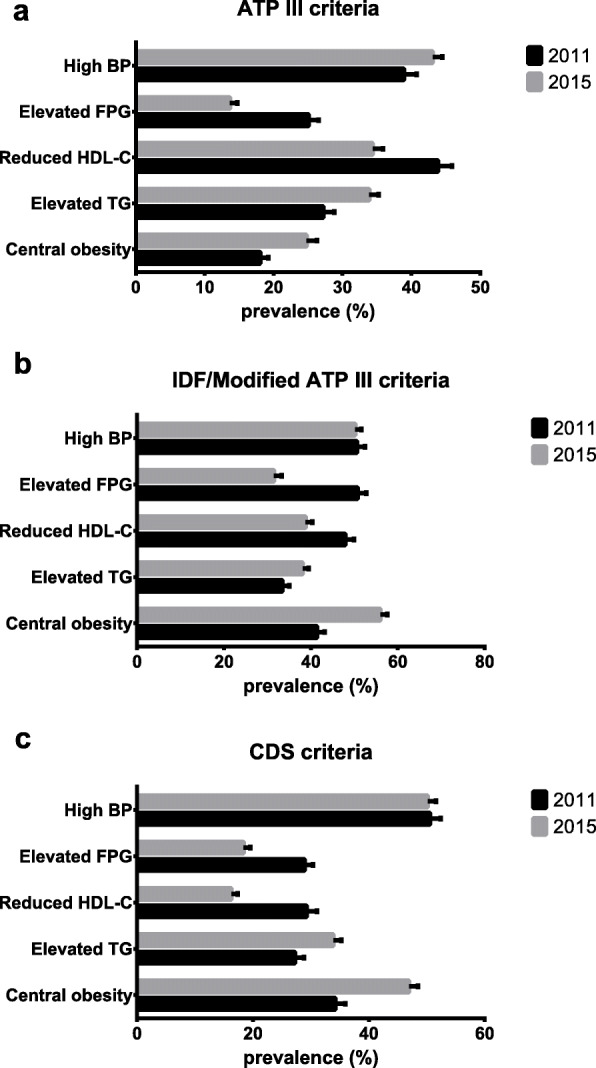
Prevalence of components of metabolic syndrome based on different definitions in 2011 and 2015. Figure a, b, and c shows prevalence of components of metabolic syndrome in 2011 and 2015 based on ATP III, IDF/revised ATP III and CDS criteria, respectively. Dark bar graph represents the baseline wave and gray bar graph represents the third wave

The prevalence of MetS components in the total study population in 2011 and 2015 is shown in Fig. 3. Although the prevalence rate of components varies widely by the different diagnostic criteria, the components are very common in China. For example, the prevalence of elevated blood pressure in 2011 was 38.93% (95% CI: 37.08–40.77%), 50.58% (95% CI: 48.74–52.43%) and 50.58% (95% CI: 48.74–52.43%) based on definitions of ATP III, IDF/revised ATP III and CDS criteria, respectively. See Additional file 1: Table S3 for details.

### Changes in the prevalence of MetS over time from 2011 to 2015

Table 4 shows the temporal changes in the total-, sex-, residence-, marital status-, age- and region-specific prevalence of MetS based on four different definitions over time from 2011 to 2015. The trends in the total-, rural-, married-, and south-central- specific prevalence decreased significantly between 2011 and 2015 based on definitions of ATP III, revised ATP III and CDS criteria. The male- specific prevalence decreased significantly between 2011 and 2015 based on definitions of ATP III and revised ATP III criteria. In contrast, the trends prevalence in the total group and many subgroups increased significantly between 2011 and 2015 based on definitions of IDF criteria.

**Table 4:** Changes in the total-, sex-, residence-, marital status-, age- and region-specific prevalence of metabolic syndrome based on different definitions over time from 2011 to 2015

2015 vs 2011	ATP III criteria		IDF criteria		Revised ATP III criteria		CDS criteria	
	OR (95% CI)	*P* value	OR (95% CI)	*P* value	OR (95% CI)	*P* value	OR (95% CI)	*P* value
Total	0.868 (0.815–0.925)	< 0.0001	1.132 (1.071–1.198)	< 0.0001	0.857 (0.813–0.904)	< 0.0001	0.902 (0.85–0.956)	0.0006
Sex								
Male	0.765 (0.684–0.855)	< 0.0001	1.297 (1.182–1.424)	< 0.0001	0.884 (0.814–0.959)	0.0031	1.003 (0.918–1.097)	0.9434
Female	0.923 (0.855–0.996)	0.0401	1.049 (0.978–1.126)	0.1805	0.838 (0.781–0.898)	< 0.0001	0.83 (0.767–0.898)	< 0.0001
Residence								
Rural	0.848 (0.78–0.921)	< 0.0001	1.074 (0.998–1.155)	0.0573	0.847 (0.791–0.907)	< 0.0001	0.887 (0.82–0.958)	0.0024
Urban	0.894 (0.811–0.984)	0.0228	1.213 (1.111–1.324)	< 0.0001	0.868 (0.798–0.945)	0.001	0.915 (0.836–1.001)	0.0532
Marital status								
Married^a^	0.875 (0.817–0.938)	0.0002	1.115 (1.049–1.186)	0.0005	0.847 (0.799–0.898)	< 0.0001	0.904 (0.848–0.964)	0.0021
Abnormal Status^b^	0.837 (0.719–0.973)	0.0208	1.226 (1.069–1.405)	0.0035	0.907 (0.798–1.031)	0.1362	0.889 (0.77–1.027)	0.1103
Age group								
45–50	0.883 (0.758–1.03)	0.1218	1.157 (1.016–1.318)	0.0275	0.897 (0.793–1.015)	0.084	0.906 (0.785–1.046)	0.1786
51–55	0.886 (0.759–1.033)	0.123	1.199 (1.045–1.375)	0.0098	0.862 (0.757–0.983)	0.0263	0.925 (0.799–1.071)	0.2954
56–60	0.855 (0.738–0.991)	0.0378	1.165 (1.021–1.329)	0.0231	0.872 (0.77–0.988)	0.0319	0.898 (0.783,1.031)	0.126
61–65	0.919 (0.791–1.068)	0.2722	1.004 (0.877–1.149)	0.9526	0.776 (0.682–0.883)	0.0001	0.857 (0.747–0.984)	0.0287
66–70	0.776 (0.648–0.93)	0.0061	1.083 (0.92–1.275)	0.3374	0.848 (0.727–0.99)	0.0368	0.927 (0.786–1.094)	0.3715
≥ 71	0.863 (0.742–1.002)	0.0539	1.164 (1.015–1.335)	0.0301	0.865 (0.762–0.983)	0.0263	0.896 (0.778–1.032)	0.1275
Region								
North-East	0.867 (0.776–0.969)	0.0117	1.101 (0.999–1.215)	0.0528	0.861 (0.785–0.945)	0.0016	0.879 (0.793–0.974)	0.0138
North	0.969 (0.827–1.134)	0.6909	1.327 (1.149–1.533)	0.0001	0.884 (0.769–1.016)	0.0813	0.961 (0.829–1.113)	0.5948
North-West	0.942 (0.753–1.177)	0.5979	1.236 (1.008–1.515)	0.0417	0.862 (0.709–1.047)	0.1349	1.07 (0.867–1.321)	0.5273
East	0.767 (0.609–0.967)	0.0248	1.292 (1.055–1.582)	0.0132	0.911 (0.754–1.1)	0.3316	0.95 (0.766–1.179)	0.6438
South-Central	0.789 (0.691–0.9)	0.0004	0.949 (0.844–1.067)	0.3792	0.771 (0.689–0.862)	< 0.0001	0.799 (0.706–0.905)	0.0004
South-West	0.908 (0.764–1.08)	0.2776	1.167 (1.002–1.36)	0.0465	0.921 (0.802–1.057)	0.2395	0.931 (0.794–1.092)	0.3798

ORs of total population were adjusted for sex, residence, marital status, age group and region; ORs of sex were adjusted for residence, marital status, age group and region; ORs of residence were adjusted for sex, marital status, age group and region; ORs of marital status were adjusted for sex, residence, age group and region; ORs of age group were adjusted for sex,residence, marital status and region; ORs of region were adjusted for sex,residence, marital status, age group; *OR* odds ratio, *CI* confidence interval; ^a^married with spouse present; ^b^married but not living with spouse temporarily for reasons such as work, separated, divorced, widowed and never married

### Changes in the prevalence of MetS components over time from 2011 to 2015

Table 5 indicates the changes in the prevalence of central obesity, elevated triglyceride (TG), reduced HDL cholesterol (HDL-C), high fasting glucose and high blood pressure between 2011 and 2015. The prevalence of central obesity and elevated triglycerides increased, and the prevalence of reduced HDL-C and high fasting glucose decreased regardless of the definitions used. However, the prevalence of high blood pressure increased based on definitions of the ATP III criteria, but did not change according to IDF, revised ATP III or CDS criteria.

**Table 5:** Changes in the prevalence of components of metabolic syndrome based on different definitions over time from 2011 to 2015

2015 vs 2011	ATP III criteria		IDF criteria/Revised ATP III criteria		CDS criteria	
	OR (95% CI)	*P* value	OR (95% CI)	*P* value	OR (95% CI)	*P* value
Central obesity	1.439 (1.347–1.537)	< 0.0001	1.721 (1.629–1.818)	< 0.0001	1.721 (1.629–1.818)	< 0.0001
Elevated TG	1.329 (1.257–1.405)	< 0.0001	1.22 (1.156–1.287)	< 0.0001	1.329 (1.257–1.405)	< 0.0001
Reduced HDL-C	0.713 (0.676–0.753)	< 0.0001	0.731 (0.693–0.77)	< 0.0001	0.501 (0.47–0.534)	< 0.0001
Elevated fasting plasma glucose	0.4 (0.374–0.427)	< 0.0001	0.358 (0.34–0.378)	< 0.0001	0.485 (0.457–0.516)	< 0.0001
High blood pressure	1.101 (1.046–1.16)	0.0003	0.978 (0.929–1.029)	0.3864	0.978 (0.929–1.029)	0.3864

ORs were adjusted for sex, residence, marital status, age group and region; *TG* triglycerides, *HDL-C* high-density lipid cholesterol, *OR* odds ratio, *CI* confidence interval

## Discussion

Our study, based on the CHARLS survey, provided an updated estimate of the national prevalence of MetS and its temporal changes among adults aged 45 years and older in China. The estimated prevalence of MetS among adults aged ≥45 years in 2015 in China was 20.41% (95% CI: 19.02–21.8%) by ATP III criteria. This prevalence was considerably higher when using IDF, revised ATP III and CDS criteria with 34.77% (95% CI: 33.12–36.42%), 39.68% (95% CI: 37.88–41.47%) and 25.55% (95% CI: 24.19–26.91%), respectively. The prevalence decreased significantly from 2011 to 2015 based on ATP III, revised ATP III and CDS criteria. However, the prevalence increased significantly from 2011 to 2015 according to the IDF criteria.

Currently, there are no uniform diagnostic criteria for MetS. Although definitions agree on the essential components including central obesity, high TG, low HDL-C, elevated blood pressure and fasting glucose, the criteria of each component varied somewhat except for the IDF and CDS criteria (Table 1). The five components are given the same weight in the ATP III, revised ATP III and CDS criteria, but the IDF criteria emphasize that the presence of abdominal obesity was necessary for diagnosis. Additionally, relatively strict criteria were used for definitions of central obesity in ATP III criteria, low HDL-C in CDS criteria, and elevated fasting glucose in IDF and revised ATP III criteria. In this study, we found that the prevalence of MetS in 2015 was lowest based on the ATP III criteria (20.41%), followed by the CDS definition (25.55%) and IDF criteria (34.77%), and highest when defined with the revised ATP III definition (39.68%). The difference in the prevalence was due to differences in the diagnostic criteria for MetS. Similarly, CSPP survey including 109,551 participants aged ≥40 years from 30 provinces in China in 2014–2015 estimated that the latest prevalence of MetS was 18.4% by the ATP III criteria, 26.9% following by the IDF criteria, and highest by the revised ATP III criteria with 34.0% [11]. The discrepancy in the prevalence of MetS based on the same diagnostic criteria between these two studies is presumably due to differences in sampling methods, participant age range, and methods of data collection. In addition, subgroup analysis suggested that the prevalence of MetS in women, older age groups, urban areas and the north was higher than that in men, younger age groups, rural areas and the south according to the different diagnostic criteria. These findings are consistent with previous studies [8, 9, 17] and menopause, unhealthy lifestyles (such as a sedentary lifestyle), decrease in physical activity, and an unhealthy diet may explain this difference in MetS prevalence.

With rapid urbanization and an aging population, many studies have found that the prevalence of MetS increased from 2000 to 2012 in China [8, 10, 18]. However, findings from the CSPP study compared with several previous nationally representative studies using the same definition (the ATP III criteria) revealed that the prevalence of MetS in 2014–2015 was higher than that in 2000–2001 but lower than the estimated prevalence in 2009–2010 [11]. Therefore, the decreasing trend in the prevalence of MetS from 2009 to 2015 in that study is consistent with our findings based on ATP III, revised ATP III and CDS criteria between 2011 and 2015. However, the only exception was that the prevalence of MetS increased significantly from 2011 to 2015 based on the IDF criteria. Changes in the prevalence of five components of MetS from 2011 to 2015 might be responsible for this contrasting report on the temporal trends in the prevalence of MetS between different diagnostic criteria. Our findings suggested that the prevalence of central obesity, elevated TG and blood pressure significantly increased from 2011 to 2015 and are consistent with previous studies [19–23]. This could explain the finding that there is an increasing trend in the prevalence of MetS between 2011 and 2015 based on definitions of IDF criteria that required abdominal obesity as an essential criterion. Moreover, our findings showed that the prevalence of reduced HDL-C and high fasting glucose decreased from 2011 to 2015 and were also supported by previous studies [21, 24]. A possible explanation is that prevention and control of non­communicable diseases became a national health strategy and that Chinese people had easier access to clinical resources following healthcare reforms in 2013. This could be an explanation for the finding that the trends in the prevalence of MetS decreased significantly between 2011 and 2015 based on the ATP III, revised ATP III and CDS criteria.

Our study has several strengths. First, the CHARLS study is a nationally representative longitudinal survey of middle-aged and elderly individuals in China, which enabled us to estimate the prevalence of MetS. Second, strict quality control assured a high quality of data collection and reliability of the findings. Third, we comprehensively described the possible temporal changes in the prevalence of MetS based on four definitions by comparing results from the same study at different time points. This study also has several limitations. First, there was a nonresponse rate of 19.5% in 2011 and 12% in 2015, which could potentially undermine the representativeness of the study samples. Second, the current follow-up time might not be long enough to reflect the changes in the prevalence of MetS. Third, this research was an ecological study, using the group as the unit of observation and analysis. We report the subgroup prevalence and performed multivariate logistic regression to control for possible confounding factors. Moreover, the age of the study population was over 40 years in this survey and may not show the full picture of MetS in China.

## Conclusions

Regardless of the metrics used, MetS is common in the general adult population in mainland China, particularly among women and elderly adults and in urban areas and the north. Notably, temporal changes in the prevalence of MetS may vary according to different criteria.

## Supplementary Information

**Additional file 1: Table S1.** Age-specific prevalence of metabolic syndrome based on different definitions in 2011 and 2015. **Table S2.** Region-specific prevalence of metabolic syndrome based on different definitions in 2011 and 2015. **Table S3.** Prevalence of components of metabolic syndrome based on different definitions in 2011 and 2015. **Supplementary information** accompanies this paper at https://doi.org/10.7910/DVN/DQ7BGK.

## Data Availability

Dataset from the China Health and Retirement Longitudinal Study (CHARLS) http://charls.pku.edu.cn/.

## Abbreviations

ATP III criteria: National Cholesterol Education Program (NCEP) Expert Panel on Detection, Evaluation, and Treatment of High Blood Cholesterol in Adults criteria
CAPI: Computer-assisted personal interview
CDC: The China Center for Disease Prevention and Control
CDS: Chinese Diabetes Society
CDS: The Chinese Diabetes Society
CHARLS: The China Health and Retirement Longitudinal Study
CHNS: The China Health and Nutrition Survey
CNCDS: The 2010 China Noncommunicable Disease Surveillance
CSPP: The China National Stroke Prevention Project
HDL-C: High-density lipoprotein cholesterol
IDF criteria: The International Diabetes Federation
InterASIA: The International Collaborative Study of Cardiovascular Disease in ASIA
MetS: Metabolic syndrome
TG: Triglyceride
WC: Waist circumference
