# Supplementary material for: Temporal trends in the prevalence of metabolic syndrome among middle-aged and elderly adults from 2011 to 2015 in China: the China health and retirement longitudinal study (CHARLS)
Source: BMC Public Health. 2021 Jun 2;21:1045. doi: 10.1186/s12889-021-11042-x (PMC8173844; doi:10.1186/s12889-021-11042-x)
Supplement: Supplementary file 1 — Additional file 1: Table S1. Age-specific prevalence of metabolic syndrome based on different definitions in 2011 and 2015. Table S2. Region-specific prevalence of metabolic syndrome based on different definitions in 2011 and 2015. Table S3. Prevalence of components of metabolic syndrome based on different definitions in 2011 and 2015. Supplementary information accompanies this paper at https://doi.org/10.7910/DVN/DQ7BGK. [file 12889_2021_11042_MOESM1_ESM.docx]

Table S1 Age-specific prevalence of metabolic syndrome based on different definitions in 2011 and 2015

|  | n | ATP III criteria | IDF criteria | Modified ATP III criteria | CDS criteria |
| --- | --- | --- | --- | --- | --- |
| 2011 |  |  |  |  |  |
| Age group (years) |  |  |  |  |  |
| 45-50 | 2472 | 16.32(14.21-18.43) | 25.31(21.82-28.8) | 34.24(30.55-37.94) | 19.89(17.49-22.3) |
| 51-55 | 1914 | 21.07(18.6-23.54) | 29.46(25.49-33.44) | 41.19(37.51-44.86) | 26.62(22.73-30.52) |
| 56-60 | 2418 | 21.9(19.32-24.48) | 28.92(25.74-32.09) | 40.68(36.87-44.49) | 26.09(23.12-29.07) |
| 61-65 | 1886 | 22.58(20-25.16) | 32.77(29.46-36.09) | 47.19(43.27-51.11) | 29.8(26.76-32.85) |
| 66-70 | 1288 | 26.33(22.72-29.94) | 36.58(30.74-42.43) | 47.82(42.67-52.97) | 33.54(27.56-39.52) |
| ≥71 | 1868 | 22.87(19.4-26.34) | 29.6(26.01-33.19) | 43.78(39.84-47.72) | 29.72(25.55-33.89) |
| *P* for trend |  | 0.0003 | 0.0176 | <0.0001 | 0.0002 |
| 2015 |  |  |  |  |  |
| Age group (years) |  |  |  |  |  |
| 45-50 | 2143 | 15.46(12.94-17.98) | 29.73(25.16-34.3) | 32.65(28.02-37.27) | 19.83(16.4-23.26) |
| 51-55 | 2092 | 19.63(16.8-22.45) | 31.89(28.88-34.9) | 36.62(33.37-39.87) | 22.4(19.62-25.18) |
| 56-60 | 2071 | 20.3(17.76-22.85) | 36.74(32.92-40.57) | 41.61(37.85-45.37) | 26.97(23.32-30.61) |
| 61-65 | 2357 | 23.48(20.38-26.59) | 38.61(35.31-41.91) | 44.33(40.99-47.67) | 30.11(26.87-33.35) |
| 66-70 | 1741 | 21.89(19.37-24.41) | 38.13(35.07-41.19) | 43.33(40.25-46.42) | 29.78(26.81-32.76) |
| ≥71 | 2609 | 22.26(19.92-24.6) | 35.02(32.52-37.51) | 41.04(38.5-43.59) | 26(23.62-28.37) |
| *P* for trend |  | 0.0036 | 0.1741 | 0.0004 | 0.001 |

Table S2 Region-specific prevalence of metabolic syndrome based on different definitions in 2011 and 2015

|  | n | ATP III criteria | IDF criteria | Modified ATP III criteria | CDS criteria |
| --- | --- | --- | --- | --- | --- |
| 2011 |  |  |  |  |  |
| Region | 11846 |  |  |  |  |
| South-West | 2074 | 15.2(13.24-17.16) | 19.73(17.26-22.2) | 29.84(26.99-32.7) | 19.47(17.18-21.76) |
| South-Central | 2661 | 19.76(16.78-22.73) | 31.48(27.05-35.91) | 43.26(37.31-49.21) | 27.97(24.14-31.8) |
| East | 3634 | 21.27(19.2-23.34) | 30.2(27.57-32.83) | 40.79(37.83-43.74) | 26.36(23.93-28.79) |
| North-West | 955 | 23.56(18.53-28.59) | 29.59(23.99-35.18) | 43.53(38.29-48.78) | 28.23(22.28-34.17) |
| North | 1675 | 28.03(24.66-31.39) | 36.96(33.02-40.9) | 52.36(48.1-56.61) | 33.21(29.65-36.76) |
| North-East | 847 | 25.26(21.42-29.11) | 29.79(25.58-33.99) | 43.2(38.54-47.87) | 26.83(22.72-30.93) |
| *P* for trend |  | 0.0682 | 0.2441 | 0.0009 | 0.017 |
| 2015 |  |  |  |  |  |
| Region | 12912 |  |  |  |  |
| South-West | 2077 | 15.37(13.4-17.34) | 24.7(21.96-27.44) | 29.42(26.7-32.14) | 18.73(16.57-20.9) |
| South-Central | 2840 | 18.25(15.24-21.27) | 31.61(28.89-34.33) | 36.43(33.56-39.31) | 23.69(21.25-26.12) |
| East | 4359 | 20.59(18.93-22.24) | 36.34(33.42-39.26) | 41.11(37.98-44.24) | 26.3(23.66-28.93) |
| North-West | 1022 | 17.86(14.58-21.15) | 35.59(31.08-40.1) | 40.92(36.13-45.72) | 24.01(20.48-27.55) |
| North | 1676 | 27.35(24.12-30.58) | 45.06(41.38-48.74) | 50.45(46.61-54.29) | 32.6(29.67-35.52) |
| North-East | 938 | 29.18(24.91-33.46) | 43.13(38.6-47.66) | 48.07(43.3-52.84) | 33.38(29.05-37.72) |
| *P* for trend |  | <0.0001 | <0.0001 | <0.0001 | <0.0001 |

Table S3 Prevalence of components of metabolic syndrome based on different definitions in 2011 and 2015

|  | ATP III criteria | IDF criteria/  Modified ATP III criteria | CDS criteria |
| --- | --- | --- | --- |
| 2011 |  |  |  |
| Central obesity | 18.06(16.88-19.24) | 41.42(39.67-43.16) | 34.25(32.56-35.94) |
| Elevated TG | 27.24(25.66-28.82) | 33.43(31.81-35.05) | 27.24(25.66-28.82) |
| Reduced HDL-C | 43.88(41.83-45.92) | 47.92(45.9-49.93) | 29.27(27.48-31.05) |
| Elevated fasting plasma glucose | 25.1(23.63-26.56) | 50.78(48.76-52.8) | 28.94(27.52-30.37) |
| High blood pressure (130/85mm Hg) | 38.93(37.08-40.77) | 50.58(48.74-52.43) | 50.58(48.74-52.43) |
| 2015 |  |  |  |
| Central obesity | 24.79(23.27-26.31) | 56(54.41-57.59) | 46.95(45.41-48.48) |
| Elevated TG | 33.88(32.53-35.24) | 38.11(36.72-39.49) | 33.88(32.53-35.24) |
| Reduced HDL-C | 34.38(32.9-35.87) | 38.76(37.21-40.31) | 16.3(15.25-17.36) |
| Elevated fasting plasma glucose | 13.66(12.59-14.73) | 31.49(29.7-33.29) | 18.38(17.19-19.56) |
| High blood pressure (130/85mm Hg) | 43.11(41.72-44.5) | 50.19(48.75-51.63) | 50.19(48.75-51.63) |

Abbreviations: TG, triglycerides; HDL-C, high-density lipid cholesterol.
